# The benefit of planned dialysis to early survival on hemodialysis versus peritoneal dialysis: a nationwide prospective multicenter study in Korea

**DOI:** 10.1038/s41598-023-33216-w

**Published:** 2023-04-13

**Authors:** Jeong-Hoon Lim, Ji Hye Kim, Yena Jeon, Yon Su Kim, Shin-Wook Kang, Chul Woo Yang, Nam-Ho Kim, Hee-Yeon Jung, Ji-Young Choi, Sun-Hee Park, Chan-Duck Kim, Yong-Lim Kim, Jang-Hee Cho

**Affiliations:** 1 Division of Nephrology, Department of Internal Medicine, School of Medicine, Kyungpook National University, Kyungpook National University Hospital, 130 Dongdeok-ro, Jung-gu, Daegu, 41944 South Korea; 2 Clinical Research Center for End Stage Renal Disease, Daegu, South Korea; 3 Department of Statistics, Kyungpook National University, Daegu, South Korea; 4 Department of Internal Medicine, Seoul National University College of Medicine, Seoul, South Korea; 5 Department of Internal Medicine, Yonsei University College of Medicine, Seoul, South Korea; 6 Department of Internal Medicine, The Catholic University of Korea College of Medicine, Seoul, South Korea; 7 Department of Internal Medicine, Chonnam National University Medical School, Gwangju, South Korea

**Keywords:** Renal replacement therapy, End-stage renal disease

## Abstract

Optimal preparation is recommended for patients with advanced chronic kidney disease to minimize complications during dialysis initiation. This study evaluated the effects of planned dialysis initiation on survival in patients undergoing incident hemodialysis and peritoneal dialysis. Patients newly diagnosed with end-stage kidney disease who started dialysis were enrolled in a multicenter prospective cohort study in Korea. Planned dialysis was defined as dialysis therapy initiated with permanent access and maintenance of the initial dialysis modality. A total of 2892 patients were followed up for a mean duration of 71.9 ± 36.7 months and 1280 (44.3%) patients initiated planned dialysis. The planned dialysis group showed lower mortality than the unplanned dialysis group during the 1st and 2nd years after dialysis initiation (1st year: adjusted hazard ratio [aHR] 0.51; 95% confidence interval [CI] 0.37–0.72; *P* < 0.001; 2nd year: aHR 0.71; 95% CI 0.52–0.98, *P* = 0.037). However, 2 years after dialysis initiation, mortality did not differ between the groups. Planned dialysis showed a better early survival rate in hemodialysis patients, but not in peritoneal dialysis patients. Particularly, infection-related mortality was reduced only in patients undergoing hemodialysis with planned dialysis initiation. Planned dialysis has survival benefits over unplanned dialysis in the first 2 years after dialysis initiation, especially in patients undergoing hemodialysis. It improved infection-related mortality during the early dialysis period.

## Introduction

The global prevalence of end-stage kidney disease (ESKD) is steadily increasing^1^. Although the mortality rate of patients with ESKD has improved, ESKD remains one of the conditions that greatly increases the risk of death^2^. Particularly, the highest mortality rate has been reported during the early period after dialysis initiation^3–6^. Several risk factors, such as old age, anemia, comorbid cardiovascular disease, and malnutrition^7–11^, are associated with greater mortality risk during the early dialysis period. Early referral to nephrologists for patients with chronic kidney disease (CKD) has been reported as a modifiable factor in reducing early mortality after dialysis initiation^12–14^.

Early referral identifies and corrects the reversible causes of CKD and delays the progression of renal insufficiency^15–17^. Additionally, patients with CKD who are referred early can receive specialized management in various areas, such as blood pressure, electrolyte levels, and cholesterol levels, resulting in reduced cardiovascular mortality^18–20^. However, Marron et al. reported that despite sufficient time, nearly half of the referred patients with CKD did not receive adequate education on renal replacement therapy options^21^. The lack of sufficient education hinders planned dialysis and increases patient complications and medical costs^21–23^.

Additionally, numerous factors can affect the timing and planning of dialysis in patients with CKD, such as asymptomatic status of advanced CKD, rapid progression of underlying kidney disease, and socioeconomic barriers^21^. Our previous study also demonstrated that not the early referral but the planned dialysis improves both quality of life and depression in newly diagnosed patients with ESKD^15^. However, there is limited information regarding the association between planned dialysis and early mortality after dialysis initiation. This study evaluated the effect of planned dialysis on the survival by period after dialysis of patients undergoing incident dialysis, and compared the effects according to the dialysis modality.

## Results

### Baseline characteristics

Among 2892 newly initiated dialysis patients, 1280 (44.3%) underwent planned dialysis. The mean age was 62.5 ± 14.2 years, and 1125 patients (38.8%) were men (Table 1). More than 50% of the patients had diabetic nephropathy as the primary renal disease. The proportion of peritoneal dialysis (PD) and early referral rate were higher in the planned dialysis group (PD: 35.2% vs. 25.3%; early referral: 58.0% vs. 53.9%; both *P* < 0.05). The proportion of patients who received nephrology care twice or more was higher in the planned dialysis group than in the unplanned group (*P* < 0.001). Among the planned dialysis group, 83.3% of patients initiated hemodialysis (HD) via native vascular access and 16.7% of patients initiated HD via synthetic vascular access. Comorbid conditions were similar in both the groups. The mean estimated glomerular filtration rate (eGFR) and urine volume at dialysis initiation did not differ between the groups. However, the proportion of anuric patients was higher in the planned dialysis group (*P* < 0.001). Hemoglobin and albumin levels were higher in the planned dialysis group. Among the socioeconomic status factors, joblessness, married status, and independent ambulation rates were higher in the planned dialysis group than in the unplanned dialysis group.

**Table 1:** Baseline characteristics of the enrolled dialysis patients.

	Planned (n = 1280)	Unplanned (n = 1612)	*P* value
Age, years	62.5 ± 13.2	62.6 ± 14.7	0.733
Sex, male n (%)	499 (39.0)	626 (38.8)	0.974
Body mass index, kg/m^2^	23.1 ± 3.3	23.0 ± 3.4	0.540
Primary renal disease, n (%)			0.509
Diabetes	706 (55.2)	895 (55.5)	
Hypertension	195 (15.2)	231 (14.3)	
Glomerulonephritis	159 (12.4)	180 (11.2)	
Others	223 (17.4)	306 (19.0)	
Dialysis type, n (%)			< 0.001
HD	830 (64.8)	1205 (74.5)	
PD	450 (35.2)	407 (25.3)	
Time from referral to dialysis, n (%)			0.029
> 12 months	742 (58.0)	869 (53.9)	
≤ 12 months	538 (42.0)	743 (46.1)	
Number of nephrology clinic visits before dialysis (n = 2764), n (%)			< 0.001
None	68 (5.6)	64 (4.2)	
1	58 (4.7)	194 (12.6)	
≥ 2	1096 (89.7)	1284 (83.3)	
Comorbidities, n (%)			
Coronary artery disease	160 (12.5)	202 (12.6)	0.963
Cerebrovascular disease	93 (7.3)	137 (8.5)	0.221
Diabetes	696 (54.4)	887 (55.1)	0.667
Congestive heart failure	117 (9.1)	167 (10.4)	0.264
Modified Charlson comorbidity index	5.2 ± 2.3	5.2 ± 2.3	0.957
Hemoglobin, g/dL	9.9 ± 1.6	9.0 ± 1.7	< 0.001
Blood urea nitrogen, mg/dL	71.1 ± 29.1	79.0 ± 39.2	< 0.001
Creatinine, mg/dL	8.4 ± 3.1	8.8 ± 4.1	0.008
eGFR, mL/min/1.73 m^2^	7.4 ± 3.1	7.6 ± 4.2	0.059
Albumin, g/dL	3.6 ± 0.5	3.3 ± 0.6	< 0.001
Calcium, mg/dL	8.2 ± 1.0	7.8 ± 1.0	< 0.001
Phosphate, mg/dL	5.1 ± 1.6	5.5 ± 2.0	< 0.001
Urine volume, mL/day	781.2 ± 574.1	806.7 ± 525.0	0.218
Urine volume < 100 mL/day, n (%)	238 (18.6)	181 (11.2)	< 0.001
Work status (n = 2754), n (%)			0.048
Jobless, including students	922 (76.5)	1134 (73.2)	
Employed	283 (23.5)	415 (26.8)	
Education (n = 2740), n (%)			0.435
< 9 years	433 (36.5)	568 (36.5)	
10–12 years	431 (36.4)	596 (38.3)	
> 13 years	321 (27.1)	391 (25.1)	
Insurance (n = 2860), n (%)			< 0.001
Medical aid covered for poor	370 (29.30)	347 (21.73)	
Medical insurance	893 (70.70)	1250 (78.27)	
Marital state (n = 2760), n (%)			0.003
Single/divorced/separated/widowed	259 (21.60)	413 (26.46)	
Married	940 (78.40)	1148 (73.54)	
Ambulation status (n = 2888), n (%)			< 0.001
Independent	1138 (89.1)	1334 (82.9)	
Partially dependent	137 (10.7)	227 (14.1)	
Dependent	3 (0.2)	49 (3.0)	
Family support (n = 2867), n (%)			0.091
None	151 (12.0)	152 (9.5)	
Partial	899 (71.2)	1184 (73.8)	
Full support	212 (16.8)	269 (16.8)	
Smoking (n = 2827), n (%)			0.695
None	701 (56.2)	881 (55.8)	
Current	125 (10.0)	174 (11.0)	
Ex-smoker	421 (33.8)	525 (33.2)	

*HD* hemodialysis, *PD* peritoneal dialysis, *eGFR* estimated glomerular filtration rate.

### Mortality rate

**Figure 1:**
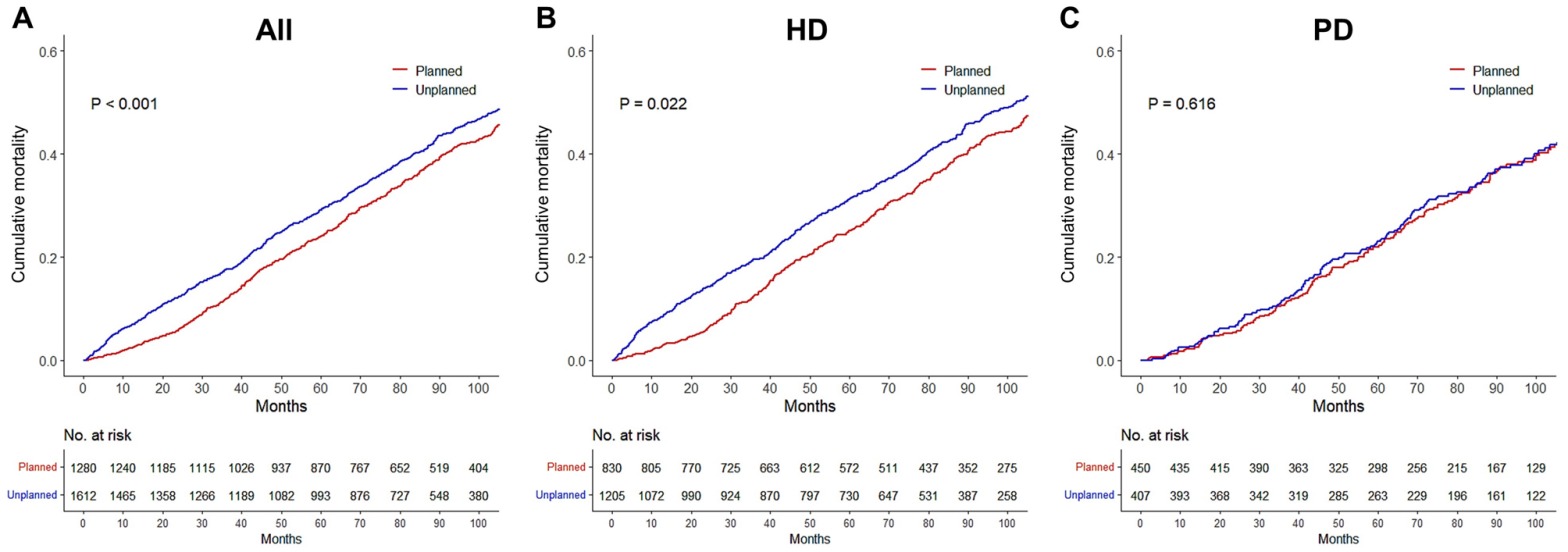
Kaplan–Meier curves for the cumulative risk of death according to planned dialysis. (**A**) All patients (**B**) Patients undergoing hemodialysis (**C**) Patients undergoing peritoneal dialysis. *HD* hemodialysis, *PD* peritoneal dialysis.

Overall, 1516 deaths (52.4%) occurred during a mean follow-up of 71.9 ± 36.7 months after initiation of dialysis. On Kaplan–Meier curve analysis, the planned dialysis group showed a better survival rate than the unplanned dialysis group during the entire observation period (log-rank *P* = 0.013; Fig. 1A). By dialysis modality, the mortality rate was significantly lower in the patients undergoing HD with planned dialysis initiation (Fig. 1B), whereas no difference was found in patients undergoing PD (Fig. 1C). The crude mortality rates in patients with planned PD, unplanned PD, planned HD, and unplanned HD were 35.1%, 36.9%, 41.2%, and 54.5%, respectively. The annual mortality rates for the period after dialysis initiation are shown in Supplementary Table S1. In both the planned and unplanned groups, the mortality rate was higher in the early period after dialysis initiation, which did not satisfy the proportional hazards assumption.

Thus, the effect on mortality was analyzed by dividing the period after dialysis into 1-year intervals (Table 2). The planned dialysis group had a decreased risk of mortality during the first 2 years after dialysis initiation in both crude and adjusted models (1st year: adjusted hazard ratio [aHR] 0.49; 95% confidence interval [CI] 0.35–0.67, *P* < 0.001; 2nd year: aHR 0.64; 95% CI 0.47–0.88, *P* = 0.005). Mortality risk did not differ between the planned and unplanned groups 2 years after dialysis (all *P* values > 0.05).

**Table 2:** Hazard ratios for annual mortality of planned dialysis by Cox proportional hazard model.

Time after dialysis, years	Type	Patient-years	No. of death	Univariate		Multivariate*	
				HR (95% CI)	*P* value	aHR (95% CI)	*P* value
0–1	Unplanned	1519.8	167	Reference		Reference	
	Planned	1257.0	53	0.38 (0.28–0.52)	< 0.001	0.51 (0.37–0.72)	< 0.001
1–2	Unplanned	1381.7	122	Reference		Reference	
	Planned	1193.7	72	0.68 (0.51–0.91)	0.010	0.71 (0.52–0.98)	0.037
2–3	Unplanned	1268.0	108	Reference		Reference	
	Planned	1109.5	95	1.01 (0.76–1.33)	0.969	0.95 (0.69–1.30)	0.739
3–4	Unplanned	1162.6	119	Reference		Reference	
	Planned	1006.5	108	1.05 (0.81–1.36)	0.720	1.02 (0.77–1.37)	0.873
4–5	Unplanned	1046.2	105	Reference		Reference	
	Planned	907.7	83	0.91 (0.68–1.22)	0.527	0.77 (056–1.06)	0.109
> 5	Unplanned	926.7	244	Reference		Reference	
	Planned	812.7	240	1.02 (0.87–1.22)	0.810	1.00 (0.82–1.23)	0.969

*Adjusted for age, sex, mCCI, serum hemoglobin, albumin, calcium, phosphate, 24-h urine volume, work status, insurance, marital status, and ambulation status.

*HR* hazard ratio, *CI* confidence interval, *aHR* adjusted hazard ratio, *mCCI* modified Charlson comorbidity index.

In the subgroup analysis by dialysis modality, the planned HD group had a decreased risk of mortality during the first 2 years after dialysis initiation in the multivariate Cox regression model (1st year: aHR 0.41; 95% CI 0.27–0.60, *P* < 0.001; 2nd year: aHR 0.61; 95% CI 0.42–0.89, *P* = 0.010) (Table 3). However, the planned PD group showed a mortality risk comparable to that of the unplanned group in all periods.

**Table 3:** Hazard ratios for annual mortality of planned dialysis by Cox proportional hazard model in hemodialysis and peritoneal dialysis.

Time after dialysis, years	Type	Hemodialysis				Peritoneal dialysis			
		Patient-years	No. of death	aHR* (95% CI)	*P* value	Patient-years	No. of death	aHR* (95% CI)	*P* value
0–1	Unplanned	1119.6	149	Reference		400.1	18	Reference	
	Planned	815.5	32	0.41 (0.27–0.62)	< 0.001	441.5	21	1.15 (0.59–2.27)	0.679
1–2	Unplanned	1007.3	94	Reference		374.5	28	Reference	
	Planned	776.7	45	0.66 (0.44–0.99)	0.045	416.9	27	0.78 (0.44–1.38)	0.393
2–3	Unplanned	925.0	74	Reference		343.0	34	Reference	
	Planned	721.4	64	0.96 (0.64–1.44)	0.849	388.1	31	0.79 (0.47–1.34)	0.384
3–4	Unplanned	853.7	79	Reference		308.9	40	Reference	
	Planned	653.5	70	1.24 (0.86–1.80)	0.248	353.0	38	0.74 (0.46–1.21)	0.229
4–5	Unplanned	770.3	81	Reference		275.9	24	Reference	
	Planned	594.7	48	0.58 (0.38–0.88)	0.010	313.1	35	1.11 (0.63–1.98)	0.715
> 5	Unplanned	683.2	180	Reference		243.5	64	Reference	
	Planned	538.2	173	1.08 (0.85–1.38)	0.535	274.5	67	0.79 (0.54–1.15)	0.221

*Adjusted for age, sex, mCCI, serum hemoglobin, albumin, calcium, phosphate, 24-h urine volume, work status, insurance, marital status, and ambulation status.

*aHR* adjusted hazard ratio, *CI* confidence interval, *mCCI* modified Charlson comorbidity index.

**Figure 2:**
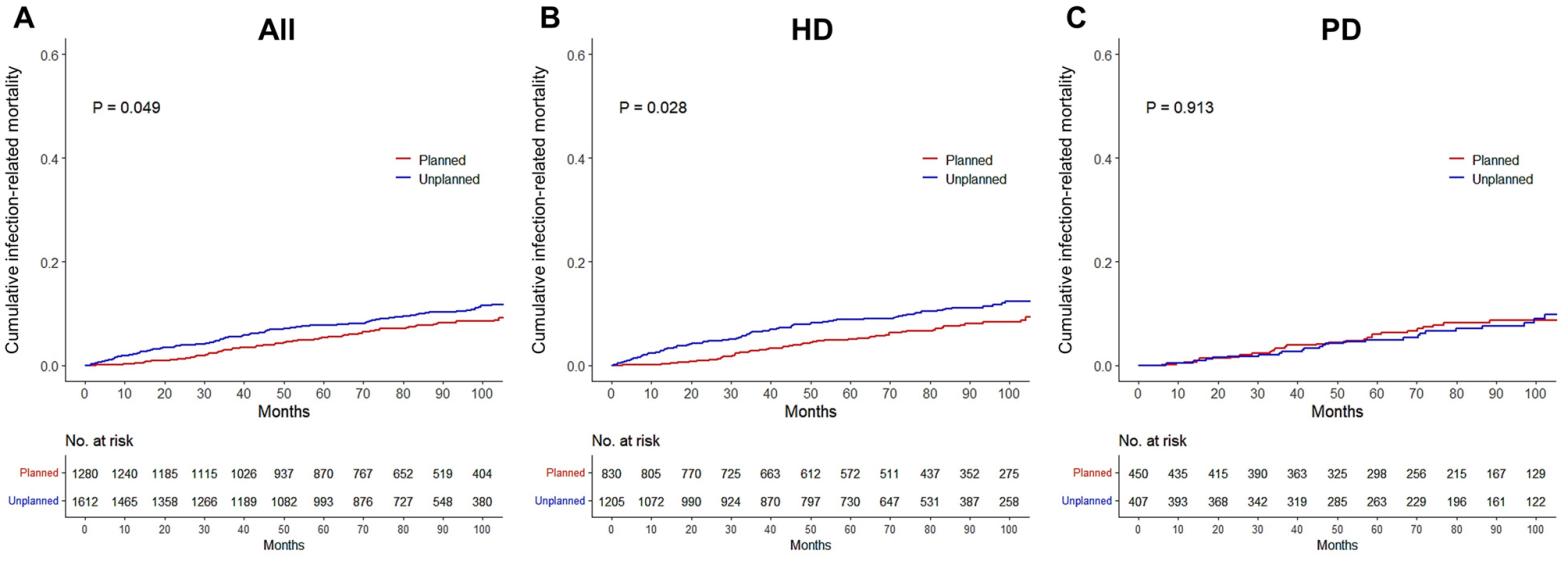
Kaplan–Meier curves for the cumulative risk of infection-related death according to planned dialysis (**A**) All patients (**B**) Patients undergoing hemodialysis (**C**) Patients undergoing peritoneal dialysis. *HD* hemodialysis, *PD* peritoneal dialysis.

Differences in infection-related mortality rates are shown in Fig. 2. Among all the patients, the planned dialysis group showed higher survival rates for infection-related deaths (Fig. 2A). The planned dialysis group had better survival for infection-related mortality only in patients undergoing HD, but not in patients undergoing PD (Fig. 2B,C). In the Cox regression model used to analyze annual infection-related mortality, the risk of infection-related death during the 1st year in the planned HD group was lower than that in the unplanned HD group (Supplementary Table S2). Planned dialysis did not cause any differences in cardiovascular mortality in all patients and dialysis subgroups (all *P* > 0.05).

### Predictors for planned dialysis

Logistic regression analysis was performed to identify the factors associated with planned dialysis (Table 4). Patients who selected PD and those who were partially dependent for ambulation or could move independently were associated with planned dialysis. Anuric patients whose urine volume was less than 100 mL/day also favored planned dialysis.

**Table 4:** Factors associated with planned dialysis by logistic regression model.

Variables	Univariate		Multivariate	
	OR (95% CI)	*P* value	aOR (95% CI)	*P* value
Age	1.00 (0.99–1.00)	0.423	1.01 (1.00–1.01)	0.122
Sex	1.00 (0.86–1.17)	0.968	1.00 (0.86–1.17)	0.984
Early referral				
≤ 12 months	Reference		Reference	
> 12 months	1.18 (1.02–1.37)	0.029	1.16 (0.99–1.34)	0.063
Work status				
Jobless including students	Reference			
Employed	0.84 (0.71–1.00)	0.051		
Dialysis type				
HD	Reference		Reference	
PD	1.61 (1.37–1.89)	< 0.001	1.61 (1.36–1.90)	< 0.001
Ambulation status				
Dependent	Reference		Reference	
Independent	13.90 (0.42–44.70)	< 0.001	12.57 (3.89–40.59)	< 0.001
Partially dependent	9.78 (2.99–31.98)	0.002	8.95 (2.73–29.34)	0.004
Urine volume				
< 100 mL/day	Reference		Reference	
≥ 100 mL/day	0.57 (0.46–0.71)	< 0.001	0.55 (0.44–0.68)	< 0.001

*OR* odds ratio, *CI* confidence interval, *aOR* adjusted odds ratio, *HD* hemodialysis, *PD* peritoneal dialysis.

## Discussion

This study demonstrated the beneficial effects of planned dialysis on improving early mortality in patients with new-onset ESKD. The first 2-year mortality improvement was clear in the planned dialysis group, while the effect decreased as the time after dialysis initiation increased. In particular, the survival advantages of planned dialysis were found in patients on HD, but not in patients on PD. This study used a nationwide prospective ESKD cohort and long-term mortality data to strengthen the results. Some patients with CKD may delay dialysis and are reluctant to prepare for it. Considering the high mortality in the early stage of dialysis, nephrologists need to educate and guide patients with CKD to prepare for timely initiation of dialysis.

The early period of CKD to ESKD is the most vulnerable period for patients undergoing incident dialysis^5,6^. Although there are differences among countries, up to 30% mortality has been reported within 1 year of dialysis initiation^2^. Among the various risk factors associated with early mortality, unplanned dialysis has the highest risk of early mortality among incident dialysis patients in the United States^4^. We confirmed that planned dialysis could effectively improve early mortality by analyzing annual mortality after dialysis initiation. Therefore, it is necessary to actively initiate planned dialysis. It can be difficult to persuade patients to undergo planned dialysis. Complex determinants of planned dialysis include different patient characteristics, medical costs for preparing dialysis, adherence to care, and underlying conditions^6^. Despite these complexities, nephrologists need to fully explain the advantages and provide patients with the opportunity for planned dialysis.

Globally, medical expenses for treating patients with ESKD have been increasing^24^. It has been confirmed that planned dialysis has the advantage of reducing medical costs and mortality. Shimizu et al. analyzed the 2-year mortality and medical costs of patients undergoing incident dialysis in Japan^25^. After adjusting for various confounding factors, planned dialysis was associated with lower mortality and medical costs. Although the health insurance system differs among countries, Japan has compulsory affiliation, free access, and a low copayment health insurance system, similar to South Korea^26^. Therefore, planned dialysis would benefit the financial stability of the long-term national healthcare system in South Korea.

Early referral to nephrologists has several benefits for patients with CKD, such as treating reversible causes of nephropathy, specialized nephrology management, and the optimal time to educate and prepare renal replacement therapy^17^. However, in our previous study, the advantages for mortality or quality of life could not be confirmed in early referral patients, including both planned and unplanned dialysis^15,27^. This may be related to the proportion of planned dialysis among the early referral patients. Our results revealed that early referral was not associated with planned dialysis, despite sufficient time. This indicates that preparation of the dialysis approach and optimal dialysis initiation are more important factors in improving mortality than early referral itself. In addition, more than half of the patients with early referral still did not receive planned dialysis, which indicates that there will be a blind spot where we need to pay attention.

Among HD and PD, patients with planned HD showed an improvement in mortality in the early period of dialysis compared to those with unplanned HD. This might be related to the increased risk of infection, such as catheter-related infections, in patients undergoing unplanned HD. The results of the subgroup analysis that showed increased infection-related mortality during the 1st year after HD support the probability of causation. Previous studies have also confirmed that HD through a temporary catheter increases the risk of infection and thrombosis^28,29^. Yap et al. reported that the prolonged duration of catheter insertion increases the risk of catheter-related bloodstream infection (CRBSI)^30^ and the mean duration from catheter insertion to first CRBSI was 6 months. Another study showed the incidence of CRBSI decreases after 9 months after catheter insertion, because of switching to HD using a vascular access. For these reasons, planned HD has an early survival benefit compared to unplanned HD, but the benefit is disappeared later. In addition, patients on PD generally have a lower infectious complication incidence than patients with HD^31^, which may affect the results that there was no difference in early mortality rate between the planned and unplanned PD groups. Therefore, education on the advantages of planned dialysis and preparation of permanent vascular access should be encouraged especially in patients willing to undergo HD.

The factors that hinder optimal initiation of dialysis include patients’ reluctance, sudden progression of kidney disease, vascular surgeons’ delay in performing vascular access, and physicians’ underestimation of CKD status^21,32,33^. Additionally, our results show that patients with CKD with better physical conditions are more likely to select planned dialysis. These patients can tolerate treatment with dialysis preparation and have a longer life expectancy. In addition, patients planning to undergo PD are more likely to select planned dialysis. This may be related to a better understanding of the necessity and options of renal replacement therapy in patients with CKD who are planning PD. In contrast, patients with CKD with a large residual urine volume tend to be reluctant to undergo planned dialysis. In these cases, patients with CKD and clinicians might decide that dialysis was not urgently needed because the symptoms of edema and uremia were not severe. A multidisciplinary approach and government support are needed to overcome these barriers and increase planned dialysis rates. A comprehensive counseling system, including dietary information, psychosocial care, economic support for medical expenses, and education on renal replacement therapy, will help increase the planned dialysis rate^15^. Interestingly, a Taiwanese research group emphasized that incentive reimbursement for healthcare providers at the government level can provide survival benefits and long-term medical cost saving for patients with CKD^34^. To improve the prognosis of patients with CKD, it is important to encourage early regular visits to a nephrologist and long-term care before dialysis^35,36^. Incentives for nephrology care will be helpful to increase the opportunity for early referral and professional care in this respect. As part of this effort, the Korean Society of Nephrology recently introduced a shared decision-making (SDM) program^37,38^. SDM can help improve patients’ knowledge of renal replacement therapy and reduce unnecessary conflicts in the decision-making process related to uncertainty regarding their worth^37^. These programs will contribute to increasing the planned dialysis rate and improving patient outcomes.

The strength of this study was that we identified the long-term mortality of dialysis patients and determined how long the advantages of planned dialysis were maintained, especially by the dialysis modality. This study had some limitations. First, this was an observational study; therefore, bias could exist between measured and unmeasured confounders. However, it is impossible to perform a randomized controlled trial on this subject owing to ethical issues. We used a nationwide ESKD cohort with a relatively large number of patients and adjusted for various possible confounding factors to reduce the bias. Second, we could not specify the reason or pathophysiological mechanism to explain the improvement in mortality from planned dialysis. Third, there may be some limitations for generalizing the study results due to Korea’s unique care system and racial characteristics. Forth, we could not investigate the effect of planned dialysis on HD or PD technical survival other than patient survival. This needs to be confirmed with future research.

In conclusion, planned dialysis has survival benefits over unplanned dialysis during the early dialysis period. The beneficial effects lasted up to 2 years after the initiation of dialysis. The advantages of planned dialysis were evident in patients undergoing HD. Optimal dialysis initiation should be recommended as a multidisciplinary approach to reduce early mortality in patients undergoing incident dialysis.

## Methods

### Study population

**Figure 3:**
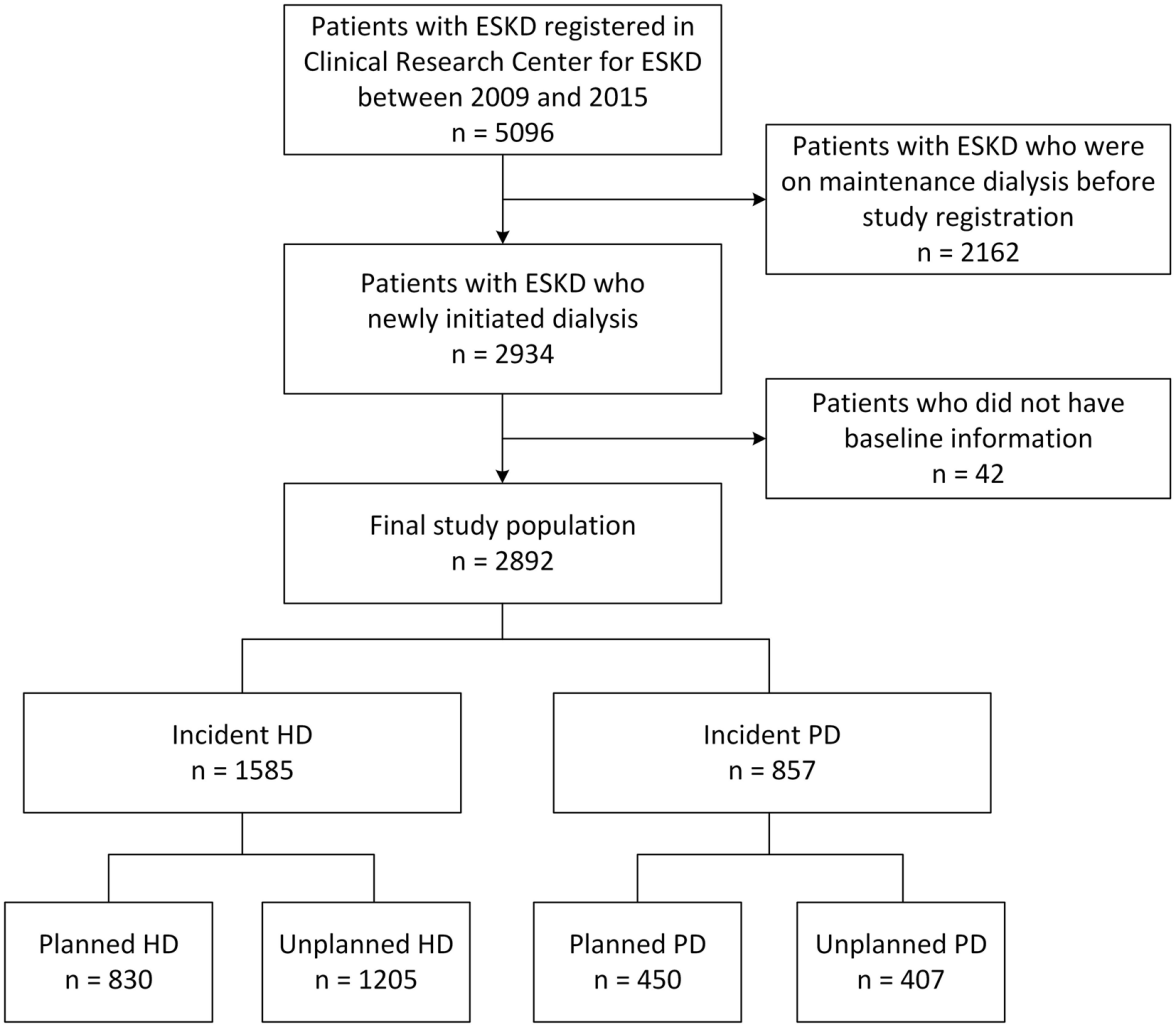
Study population. *ESKD* end-stage kidney disease, *HD* hemodialysis, *PD* peritoneal dialysis.

This study analyzed patient data from the Clinical Research Center for End-Stage Renal Disease (CRC for ESRD) in Korea. The CRC for ESRD cohort is a nationwide, multicenter, web-based, prospective cohort of patients with ESKD undergoing dialysis in 31 hospitals in South Korea (NCT00931970). The details of the study cohort have been previously described^39^. Between July 2009 and June 2015, a total of 2892 patients aged > 20 years and newly diagnosed with ESKD who underwent maintenance dialysis for 3 months were enrolled and analyzed (Fig. 3). The medical ethics committees of all participating dialysis centers approved the CRC registry for ESRD, and informed consent was obtained from all patients before inclusion.

### Data collection

All cohort data were registered in the web-based CRC for ESRD database, and data related to this study were selectively extracted. Baseline patient information, including age, sex, height, weight, body mass index, primary renal disease, dialysis type, comorbid diseases, modified Charlson comorbidity index (mCCI), and laboratory data, was collected at enrollment. The mCCI was calculated for each patient at initiation of dialysis^40,41^, and the eGFR was calculated using the CKD-Epidemiology Collaboration equation^42,43^. Patient information associated with socioeconomic status, including work status, education, ambulation status, and family support, was also collected at enrollment. Planned dialysis was defined as dialysis therapy initiated with permanent access to and maintenance of the initial dialysis modality for at least 90 days. Dialysis modality was defined as the modality maintained 90 days after dialysis initiation or at dialysis initiation if death occurred within 90 days.

### Outcomes

The main outcome was all-cause mortality after dialysis initiation. Mortality data, including the cause of death in cohort patients up to December 2019 were obtained from Statistics Korea, and patients were censored at the time of kidney transplantation. Analyses according to the cause of death and dialysis modality were also performed.

### Statistical analysis

Continuous variables were presented as mean ± standard deviation, and categorical variables were presented as numbers and percentages. Student’s t-test was used to compare continuous variables between the planned and unplanned dialysis groups, and Pearson’s chi-square test or Fisher’s exact test was used to compare distributions of categorical variables between groups, as appropriate. Kaplan–Meier curves and log-rank test were used to compare the differences in mortality between the planned and unplanned dialysis groups. The association between planned dialysis and mortality according to time after dialysis was determined using multivariable Cox proportional hazard regression models. Adjustment factors were selected as clinically relevant variables, including age, sex, mCCI, 24-h urine volume, work status, insurance, marital status, and ambulation status. The proportional hazards assumption was tested using graphical and weighted residual analyses. Weighted residual analyses demonstrated that the proportional hazards assumption was violated, and we fitted separate models for annual intervals. Factors associated with planned dialysis were evaluated using a logistic regression analysis. Factors that differed in their baseline characteristics were selected in the univariable analysis. Variables with significance, including age and sex, were included in the multivariate analysis. Statistical analyses were performed using SPSS version 22.0 (IBM Corp., Armonk, NY, USA) and R (R Foundation for Statistical Computing, Vienna, Austria; www.r-project.org). *P* values < 0.05 were considered statistically significant.

### Ethics declarations

The data do not contain personal information and do not infringe on the privacy of patients. This study was approved by the Institutional Review Board of the Kyungpook National University Hospital (2011-01-041). All patients provided written informed consent before participation, and the study was conducted according to the tenets of the 2013 Declaration of Helsinki.

## Supplementary Information

Supplementary Information.

## Data Availability

The data that support the findings of this study are available on reasonable request from the corresponding authors. The data are not publicly available due to privacy or ethical restriction.
