# Supplementary material for: The benefit of planned dialysis to early survival on hemodialysis versus peritoneal dialysis: a nationwide prospective multicenter study in Korea
Source: Sci Rep. 2023 Apr 13;13:6049. doi: 10.1038/s41598-023-33216-w (PMC10102303; doi:10.1038/s41598-023-33216-w)
Supplement: Supplementary file 1 — Supplementary Information. [file 41598_2023_33216_MOESM1_ESM.docx]

**The benefit of planned dialysis to early survival on hemodialysis versus peritoneal dialysis: a nationwide prospective multicenter study in Korea**

Jeong-Hoon Lim^1,2¶^, Ji Hye Kim^1,2¶^, Yena Jeon^2,3^, Yon Su Kim^2,4^, Shin-Wook Kang^2,5^, Chul Woo Yang^2,6^, Nam-Ho Kim^2,7^, Hee-Yeon Jung^1,2^, Ji-Young Choi^1,2^, Sun-Hee Park^1,2^, Chan-Duck Kim^1,2^, Yong-Lim Kim^1,2*^, Jang-Hee Cho^1,2*^

^1^Division of Nephrology, Department of Internal Medicine, School of Medicine, Kyungpook National University, Kyungpook National University Hospital, Daegu, South Korea

^2^Clinical Research Center for End Stage Renal Disease, Daegu, South Korea

^3^Department of Statistics, Kyungpook National University, Daegu, South Korea

^4^Department of Internal Medicine, Seoul National University College of Medicine, Seoul, South Korea

^5^Department of Internal Medicine, Yonsei University College of Medicine, Seoul, South Korea

^6^Department of Internal Medicine, The Catholic University of Korea College of Medicine, Seoul, South Korea

^7^Department of Internal Medicine, Chonnam National University Medical School, Gwangju, South Korea

^¶^These authors contributed equally to this work.

^*^**Corresponding Authors**

**Supplementary Material Table of Contents**

**Supplementary Table S1.** Mortality rates per year after initiation of dialysis

**Supplementary Table S2.** Hazard ratios for annual infection-related mortality of planned dialysis using Cox proportional hazard model for hemodialysis and peritoneal dialysis

**Supplementary Information.** STROBE checklist

**Supplementary Table S1.** Mortality rates per year after initiation of dialysis

|  | Planned dialysis | | | | |  | Unplanned dialysis | | | | |
| --- | --- | --- | --- | --- | --- | --- | --- | --- | --- | --- | --- |
| Time after dialysis, years | All-cause death, n | Cardiovascular death, n | Infection-related death, n | No. at risk | Deaths/1000 persons/year | | All-cause death, n | Cardiovascular death, n | Infection-related death, n | No. at risk | Deaths/1000 persons/year |
| 0 to 1 | 53 | 8 | 4 | 1280 | 42.17 | | 167 | 31 | 32 | 1612 | 109.88 |
| 1 to 2 | 72 | 15 | 11 | 1227 | 29.74 | | 122 | 34 | 25 | 1445 | 43.16 |
| 2 to 3 | 95 | 35 | 21 | 1155 | 27.78 | | 108 | 30 | 21 | 1323 | 27.59 |
| 3 to 4 | 108 | 41 | 13 | 1060 | 25.80 | | 119 | 39 | 20 | 1215 | 24.75 |
| 4 to 5 | 83 | 35 | 10 | 952 | 17.60 | | 105 | 25 | 9 | 1096 | 19.34 |
| > 5 | 240 | 67 | 34 | 869 | 2.85 | | 244 | 50 | 29 | 991 | 2.62 |

**Supplementary Table S2.** Hazard ratios for annual infection-related mortality of planned dialysis using the Cox proportional hazard model for hemodialysis and peritoneal dialysis

|  |  |  | Hemodialysis |  |  |  | Peritoneal dialysis |  |
| --- | --- | --- | --- | --- | --- | --- | --- | --- |
| Time after dialysis, years | Type | No. at risk | aHR^*^ (95%CI) | *P* value |  | No. at risk | aHR^*^ (95%CI) | *P* value |
| 0 to 1 | Unplanned | 1205 | Reference |  |  | 407 | Reference |  |
|  | Planned | 830 | 0.07 (0.01–0.54) | 0.010 |  | 450 | 4.30 (0.37–49.99) | 0.244 |
| 1 to 2 | Unplanned | 1056 | Reference |  |  | 389 | Reference |  |
|  | Planned | 798 | 0.42 (0.16–1.07) | 0.069 |  | 429 | 0.38 (0.08–1.79) | 0.219 |
| 2 to 3 | Unplanned | 962 | Reference |  |  | 361 | Reference |  |
|  | Planned | 753 | 0.60 (0.27–1.32) | 0.205 |  | 402 | 5.50 (0.64–46.99) | 0.119 |
| 3 to 4 | Unplanned | 888 | Reference |  |  | 327 | Reference |  |
|  | Planned | 689 | 1.05 (0.43–2.30) | 0.909 |  | 371 | 0.43 (0.08–2.36) | 0.334 |
| 4 to 5 | Unplanned | 809 | Reference |  |  | 287 | Reference |  |
|  | Planned | 619 | 0.62 (0.18–2.22) | 0.466 |  | 333 | 2.21 (0.42–11.75) | 0.351 |
| > 5 | Unplanned | 728 | Reference |  |  | 263 | Reference |  |
|  | Planned | 571 | 0.71 (0.30–1.70) | 0.439 |  | 298 | 9.63 (0.52–179.14) | 0.129 |

^*^Adjusted for age, sex, mCCI, serum hemoglobin, albumin, calcium, phosphate, 24-hour urine volume, work status, insurance, marital status, and ambulation status.

Abbreviations: aHR, adjusted hazard ratio; CI, confidence interval; mCCI, modified Charlson comorbidity index.

**Supplementary Information.** STROBE checklist

**Modified STROBE Statement—checklist of items that should be included in reports of observational studies (Cohort/Cross-sectional and case-control studies)**

|  | Item No | Recommendation |  |  |
| --- | --- | --- | --- | --- |
| **Title and abstract** | 1 | (*a*) Indicate the study’s design with a commonly used term in the title or the abstract |  | Page 3 |
|  |  | (*b*) Provide in the abstract an informative and balanced summary of what was done and what was found |  | Page 3 |
| Introduction | | |  |  |
| Background/rationale | 2 | Explain the scientific background and rationale for the investigation being reported |  | Page 4 |
| Objectives | 3 | State specific objectives, including any prespecified hypotheses |  | Page 4 |
| Methods | | |  |  |
| Study design | 4 | Present key elements of study design early in the paper |  | Page 11 |
| Setting | 5 | Describe the setting, locations, and relevant dates, including periods of recruitment, exposure, follow-up, and data collection |  | Page 11,12 |
| Participants | 6 | (*a*) *Cohort study*—Give the eligibility criteria, and the sources and methods of selection of participants. Describe methods of follow-up  *Case-control study*—Give the eligibility criteria, and the sources and methods of case ascertainment and control selection. Give the rationale for the choice of cases and controls  *Cross-sectional study*—Give the eligibility criteria, and the sources and methods of selection of participants |  | Page 11 |
| Variables | 7 | Clearly define all outcomes, exposures, predictors, potential confounders, and effect modifiers. Give diagnostic criteria, if applicable |  | Page 12 |
| Data sources/ measurement | 8* | For each variable of interest, give sources of data and details of methods of assessment (measurement). |  | Page 11,12 |
| Bias | 9 | Describe any efforts to address potential sources of bias |  | 12,13 |
| Study size | 10 | Explain how the study size was arrived at (if applicable) |  | N/A |
| Quantitative variables | 11 | Explain how quantitative variables were handled in the analyses. If applicable, describe which groupings were chosen and why |  | Page 12,13 |
| Statistical methods | 12 | (*a*) Describe all statistical methods, including those used to control for confounding |  | Page 12,13 |
|  |  | (*b*) Describe any methods used to examine subgroups and interactions |  | Page 12,13 |
|  |  | (*c*) Explain how missing data were addressed |  | Page 12,13 |
|  |  | (*d*) *Cohort study*—If applicable, explain how loss to follow-up was addressed  *Case-control study*—If applicable, explain how matching of cases and controls was addressed  *Cross-sectional study*—If applicable, describe analytical methods taking account of sampling strategy |  | Page 12,13 |
|  |  | (*e*) Describe any sensitivity analyses |  | N/A |
| Results | | |  |  |
| Participants | 13* | (a) Report numbers of individuals at each stage of study—eg numbers potentially eligible, examined for eligibility, confirmed eligible, included in the study, completing follow-up, and analyzed |  | Page 5 |
|  |  | (c) Use of a flow diagram |  | Figure3 |
| Descriptive data | 14* | (a) Give characteristics of study participants (eg demographic, clinical, social) and information on exposures and potential confounders |  | Page 5 |
|  |  | (b) Indicate number of participants with missing data for each variable of interest |  | Page 5, Table1 |
|  |  | (c) *Cohort study*—Summarise follow-up time (eg, average and total amount) |  | Page 5 |
| Outcome data | 15* | *Cohort study*—Report numbers of outcome events or summary measures over time |  | Page 5,6 |
|  |  | *Case-control study—*Report numbers in each exposure category, or summary measures of exposure |  |  |
|  |  | *Cross-sectional study—*Report numbers of outcome events or summary measures |  |  |
| Main results | 16 | (*a*) Give unadjusted estimates and, if applicable, confounder-adjusted estimates and their precision (eg, 95% confidence interval). Make clear which confounders were adjusted for and why they were included |  | Page 5,6 |
| Other analyses | 17 | Report other analyses done—eg analyses of subgroups and interactions, and sensitivity analyses |  | Page 5,6,7 |
| Discussion | | |  |  |
| Key results | 18 | Summarise key results with reference to study objectives |  | Page 10,11 |
| Limitations | 19 | Discuss limitations of the study, taking into account sources of potential bias or imprecision. Discuss both direction and magnitude of any potential bias |  | Page 10,11 |
| Interpretation | 20 | Give a cautious overall interpretation of results considering objectives, limitations, multiplicity of analyses, results from similar studies, and other relevant evidence |  | Page 7-11 |
| Generalisability | 21 | Discuss the generalisability (external validity) of the study results |  | Page 11 |

*Give information separately for cases and controls in case-control studies and, if applicable, for exposed and unexposed groups in cohort and cross-sectional studies.

**Note:** An Explanation and Elaboration article discusses each checklist item and gives methodological background and published examples of transparent reporting. The STROBE checklist is best used in conjunction with this article (freely available on the Web sites of PLoS Medicine at http://www.plosmedicine.org/, Annals of Internal Medicine at http://www.annals.org/, and Epidemiology at http://www.epidem.com/). Information on the STROBE Initiative is available at www.strobe-statement.org.
